# Resetting immunometabolic set points in autoimmune disease

**DOI:** 10.1016/j.jtauto.2026.100391

**Published:** 2026-08-01

**Authors:** Xiao Yu, Yidan Zhang, Anquan Shang

**Affiliations:** a Department of Otolaryngology-Head and Neck Surgery, Shanghai Sixth People's Hospital Affiliated to Shanghai Jiao Tong University School of Medicine, Shanghai, China; b ENT Institute and Department of Otorhinolaryngology, Eye & ENT Hospital, Fudan University, Shanghai, China; c Department of Respiratory and Critical Care Medicine, Shanghai Chest Hospital, Shanghai Jiao Tong University School of Medicine, Shanghai, China; d Department of Laboratory Medicine, Affiliated Lianyungang Clinical College of Nantong University, Lianyungang, China

**Keywords:** Autoimmune disease, Immunometabolism, Immunometabolic set point, Metabolic memory, Therapeutic reset, Immune tolerance

## Abstract

Autoimmune diseases often persist despite effective suppression of overt inflammation, and many patients experience relapse after treatment tapering or withdrawal. This clinical pattern raises the possibility that disease activity is influenced not only by ongoing immune stimulation, but also by relatively stable biological states that preserve inflammatory potential. In this Review, we propose immunometabolic set points as an integrative framework for examining how immune-cell metabolic programs, tissue metabolic niches, metabolite signaling, and immune or metabolic memory may interact in chronic autoimmune disease. Rather than representing a single pathway or biomarker, an immunometabolic set point is proposed to describe a potentially reversible multicompartment state shaped by immune and tissue interactions. Experimental studies support important roles for cellular metabolism, local nutrient and oxygen conditions, mitochondrial stress, stromal activation, and metabolites such as lactate, succinate, and itaconate in regulating immune function. However, their integration into a unified disease-maintaining state has not been directly established. We therefore distinguish evidence-supported mechanisms from broader conceptual inferences concerning relapse-prone remission and therapeutic reset. We further discuss how longitudinal single-cell profiling, spatial omics, metabolomics, and metabolic flux analysis may be used to test the framework and determine whether treatment produces transient inflammatory suppression or more durable biological reconfiguration.

## 1 Introduction

Autoimmune diseases are traditionally viewed as disorders of self-tolerance, in which defects in immune recognition and regulation permit persistent pathogenic immune activation [1,2]. This framework has enabled a broad therapeutic armamentarium, including cytokine blockade, immune-cell depletion, co-stimulation modulation and targeted inhibition of intracellular signaling pathways [[3], [4], [5]]. Yet durable disease control remains difficult to achieve. Relapse after treatment tapering or withdrawal has been documented in autoimmune diseases, including rheumatoid arthritis and systemic lupus erythematosus, whereas persistent or recurrent disease may occur despite therapies directed at apparently relevant inflammatory pathways [4,6,7]. These patterns suggest that disease activity is not determined only by the intensity of inflammation at a given moment, but also by the stability of the biological state from which that inflammation emerges.

Immunometabolism offers one way to examine this state stability. Immune cells do not use metabolic pathways simply to generate energy; glycolysis, oxidative phosphorylation, fatty acid metabolism, amino acid metabolism and mitochondrial function actively shape immune-cell activation, differentiation, survival and effector function. These pathways are regulated by nutrient and oxygen availability and by signaling nodes such as mTOR, AMPK and HIF-1α [[8], [9], [10]]. In autoimmune disease, chronic antigenic stimulation, inflammatory cytokines and tissue damage may impose metabolic demands that reinforce pathogenic immune phenotype [11]. Autoreactive T cells, B cells, plasmablasts, myeloid cells and regulatory T cells are therefore not merely activated or inactive; they occupy metabolic states that influence how they respond to antigen, tissue-derived cues and therapy [[12], [13], [14]].

A purely cell-intrinsic view, however, is insufficient. Autoimmune inflammation occurs within tissues that have their own metabolic architecture [11]. Inflamed synovium, gut mucosa, kidney, skin and central nervous system lesions differ in oxygen tension, nutrient availability, stromal activity, lipid handling, mitochondrial stress and barrier function [15]. These conditions can shape the metabolism of infiltrating immune cells, while immune cells in turn remodel the tissue environment. Metabolites such as lactate, succinate, itaconate, acetyl-CoA, kynurenine and lipid mediators further blur the boundary between metabolism and signaling [[16], [17], [18]]. They may report local stress, alter immune-cell behavior and, in some settings, contribute to chromatin remodeling or altered antigenicity.

Despite these advances, existing immunometabolic research has largely examined cellular metabolic reprogramming, tissue metabolic environments, metabolite signaling, and inflammatory memory as related but partly separate processes. These perspectives have provided important mechanistic insights, but they have not yet established how abnormalities across immune and tissue compartments may interact over time to stabilize a disease-prone state. In particular, an unresolved question is why clinical remission may remain biologically unstable after overt inflammation has subsided and why apparently effective suppression does not always translate into durable treatment-free remission.

To address this gap, we propose the immunometabolic set point as an integrative and testable framework for considering the stability of autoimmune disease states. The term refers to a relatively stable, but potentially reversible, multicompartment state arising from reciprocal interactions among immune-cell metabolic programs, tissue metabolic niches, metabolite signaling, and immune or metabolic memory. It is not intended to describe a single metabolic pathway or fixed physiological variable, nor to replace established concepts such as metabolic reprogramming, tissue immunometabolism, trained immunity, disease tolerance, or immune reset. Rather, the framework considers whether processes represented by these paradigms become coupled across cellular, tissue, and temporal scales and thereby contribute to the preservation of inflammatory potential during apparent clinical remission.

The distinctive focus of the immunometabolic set point framework is therefore biological state stability. It shifts the unit of analysis from an isolated pathway, metabolite, or immune-cell population to the feedback relationships among immune cells, non-immune tissue cells, local metabolic conditions, and memory mechanisms. It also links active disease, relapse-prone remission, and therapeutic response within a common state-transition model. Because this integrated framework has not yet been directly validated, it should be regarded as a conceptual model that organizes available evidence and generates testable predictions rather than as an established causal description of autoimmune disease.

The objective of this Review is to define the immunometabolic set point framework, distinguish it from adjacent paradigms, and examine its potential relevance to disease persistence, relapse-prone remission, and therapeutic reset in autoimmune disease. We first discuss the cellular metabolic circuits and tissue niches that may stabilize pathogenic or tolerogenic immune states. We then consider how metabolite signaling and immune or metabolic memory may connect tissue stress with persistent susceptibility to reactivation. Finally, we examine how longitudinal single-cell profiling, spatial technologies, metabolomics, and metabolic flux analysis may be used to test the framework, distinguish transient inflammatory suppression from durable biological reset, and support more precise therapeutic stratification.

## 2 Literature search strategy

A narrative literature search was conducted using PubMed and Web of Science from database inception to June 10, 2026. Search terms included combinations of “autoimmune disease,” “immunometabolism,” “metabolic reprogramming,” “immune reset,” “trained immunity,” “disease tolerance,” “tissue immunometabolism,” “metabolic memory,” “relapse,” and “remission,” together with terms related to immune-cell metabolism, tissue metabolic niches, metabolite signaling, and emerging metabolic profiling technologies. Titles and abstracts were screened for relevance to the conceptual scope of this Review. Priority was given to mechanistic and translational studies, human data where available, recent high-quality reviews, and landmark studies necessary to establish the development of the field. References were selected according to their relevance to the proposed framework rather than through formal systematic-review procedures. Because the present article was designed as a narrative review intended to develop an integrative conceptual framework, no quantitative evidence synthesis or formal risk-of-bias assessment was performed, consistent with the methodological distinction between narrative and systematic medical reviews described in contemporary guidance [19].

## 3 Defining immunometabolic set points and their conceptual boundaries

The term immunometabolic set point is used here as a conceptual framework rather than as a claim that autoimmune diseases are governed by a single fixed metabolic variable. In physiology, a set point describes a state maintained around a relatively stable range through feedback regulation [20]. By analogy, an immunometabolic set point refers to a relatively stable, but potentially reversible, multicompartment state generated by interactions among immune-cell metabolic programs, tissue metabolic conditions, metabolite signaling, and immune or metabolic memory. The framework therefore focuses on the stability of an integrated biological state rather than on any single pathway, metabolite, or cell population.

This concept is broader than metabolic reprogramming, which primarily describes changes in substrate use, bioenergetic pathways, or mitochondrial function within individual cells [8,11]. Such changes may contribute to a set point, but they do not alone define a disease-level state. It is also distinct from tissue immunometabolism, which examines metabolic interactions between immune and non-immune cells within local tissue environments [11,15]. Tissue immunometabolism provides the spatial context for the proposed framework, whereas the set point concept additionally emphasizes longitudinal stability, persistence during remission, and transitions between pathogenic and tolerogenic states.

Trained immunity represents one possible memory mechanism within an immunometabolic set point. It refers to persistent metabolic and epigenetic reprogramming of innate immune cells or their progenitors [21], but does not encompass adaptive immune memory, regulatory-cell fitness, stromal states, or persistent tissue metabolite gradients. Disease tolerance, by contrast, concerns the limitation of tissue damage and preservation of function without necessarily removing the underlying pathogenic stimulus [22]. It therefore addresses the consequences of disease for host fitness, whereas the set point framework addresses the stability of the biological state that influences whether autoimmune inflammation resolves, persists, or recurs.

The framework also differs from immune reset. Immune reset generally describes a therapeutic process or outcome in which pathogenic immune architecture is durably altered and sustained remission becomes possible [23]. An immunometabolic set point instead describes the biological state before, during, or after treatment. Within this framework, immunometabolic reset represents a transition from a pathogenic or relapse-prone set point toward a more stable tolerogenic state. Profound immune-depleting or cellular therapies may provide opportunities to study such transitions, although durable remission alone does not establish that immunometabolic reset has occurred [24].

The proposed framework also extends beyond a biomarker signature. A cytokine, metabolite, transcriptional score, or immune-cell frequency may reflect disease activity or relapse risk without defining the interacting mechanisms that stabilize the disease state [23]. An immunometabolic set point should therefore be inferred from coordinated and longitudinal changes across immune-cell function, tissue conditions, metabolite signaling, and memory compartments rather than from a single marker.

**Fig. 1:**
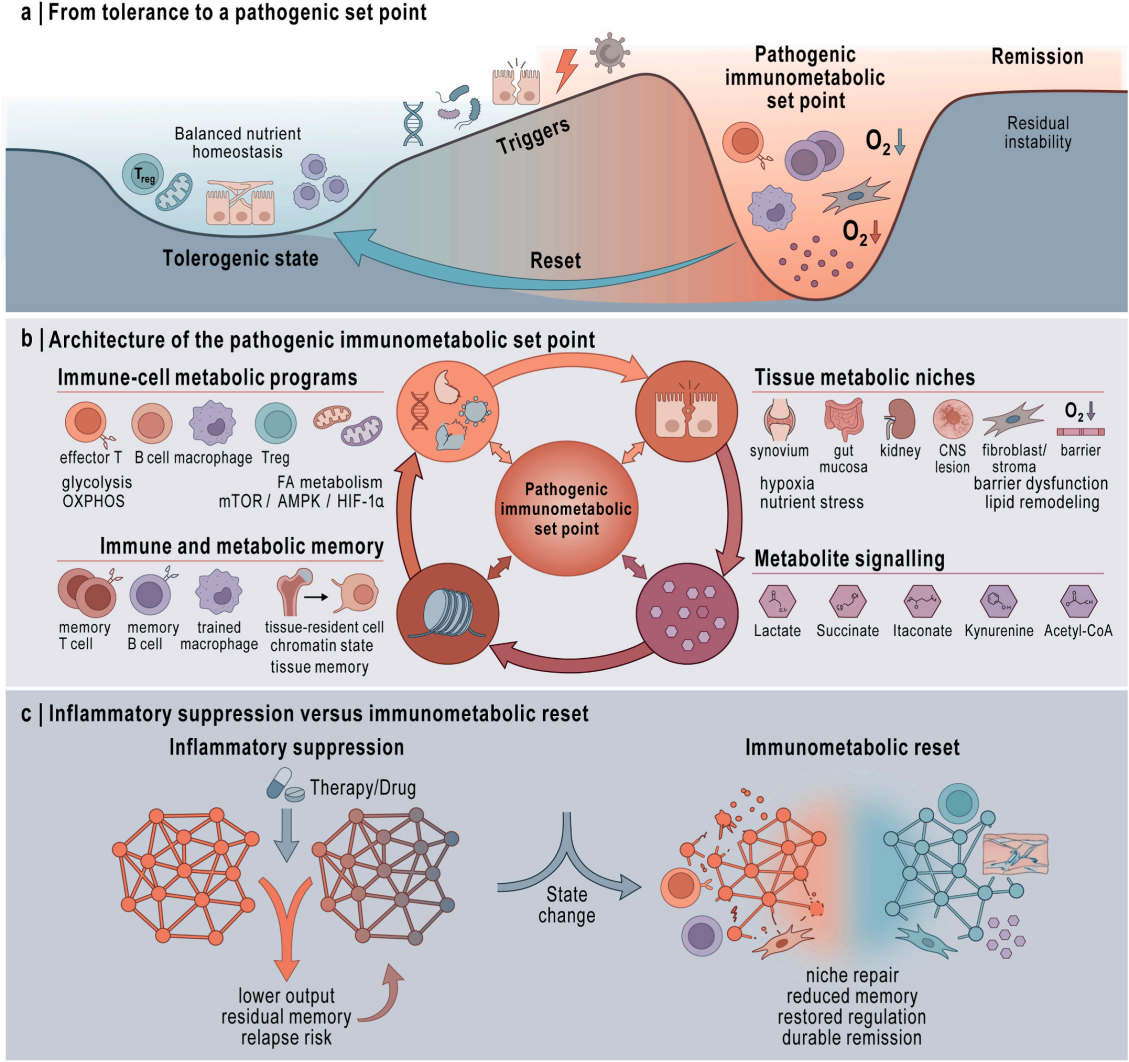
Proposed multicompartment immunometabolic set point model linking autoimmune disease persistence, remission, and reset. (a) Genetic, environmental, infectious, or barrier-related triggers may shift a tolerogenic state toward a relatively stable pathogenic immunometabolic state; clinical remission may occur while residual biological instability persists. (b) The proposed state integrates reciprocal interactions among immune-cell metabolic programs, tissue metabolic niches, metabolite signaling, and immune or metabolic memory. (c) Inflammatory suppression reduces disease output but may leave relapse-relevant processes incompletely altered, whereas evidence consistent with immunometabolic reset would include coordinated and durable reconfiguration toward tolerogenic regulation. The model is conceptual, and the relative contribution of each component is expected to vary across diseases. Acetyl-CoA, acetyl coenzyme A; AMPK, AMP-activated protein kinase; CNS, central nervous system; FA, fatty acid; HIF-1α, hypoxia-inducible factor 1 alpha; mTOR, mechanistic target of rapamycin; OXPHOS, oxidative phosphorylation; Treg, regulatory T cell.

The principal conceptual advance lies in integrating these previously studied processes into a dynamic model of disease-state stability. First, the framework shifts the unit of analysis from an isolated pathway or cell population to a multicompartment immune–tissue state. Second, it links active disease, residual biological instability during clinical remission, relapse susceptibility, and therapeutic reset within a common state-transition model. Third, it generates testable predictions: patients with similar clinical activity may have different dominant set point drivers; relapse-prone remission may retain abnormalities across several compartments; and durable reset should be associated with coordinated and persistent changes in immune, tissue, metabolic, and memory states. Longitudinal single-cell profiling, spatial analysis, metabolomics, and metabolic flux measurements may help evaluate these predictions [15,25].The proposed multicompartment model and its relationship to disease persistence, relapse-prone remission, inflammatory suppression, and immunometabolic reset are summarized in Fig. 1.

Evidence supporting individual cellular, metabolic, and tissue-level mechanisms is substantially stronger than evidence supporting their integration into a unified disease-maintaining state. Accordingly, observations demonstrated in experimental or clinical studies are distinguished throughout this Review from broader inferences concerning cross-compartment interactions, relapse susceptibility, and therapeutic reset. These broader propositions should currently be regarded as testable hypotheses generated by the immunometabolic set point framework rather than as an established causal architecture of autoimmune disease.

## 4 Cellular circuits that may contribute to pathogenic immune persistence

Immune-cell metabolism is often summarized as a switch between glycolysis and oxidative phosphorylation, but this shorthand is too simple for autoimmune disease. Experimental studies indicate that metabolic circuits can support immune-cell activation, survival, migration, cytokine production, antigen presentation, and tissue adaptation, and may thereby contribute to the persistence of pathogenic immunity. These circuits vary across cell types and disease contexts, and the same pathway may have different consequences depending on timing, tissue environment and differentiation state. Within the proposed framework, the relevant question is not only whether a pathway is increased or decreased, but whether it contributes to the relative stability of a pathogenic or tolerogenic immune state.

Effector T cells illustrate this principle. Antigen recognition, co-stimulation and inflammatory cytokines increase the demand for energy production and biosynthesis [12]. Glycolysis, glutamine metabolism, mitochondrial activity and mTOR-dependent nutrient sensing can support clonal expansion, cytokine production and differentiation toward inflammatory phenotypes [12]. In autoimmune disease, these programs may sustain tissue-infiltrating T cells and T follicular helper cells that provide help to autoreactive B cells [26]. Yet T-cell metabolism is not determined by activation signals alone. Nutrient availability, hypoxia, stromal interactions and competition with other immune populations influence which metabolic programs are available and which are favored. A pathogenic T-cell state is therefore partly cell autonomous and partly imposed by the tissue in which the cell operates [11].

Regulatory T cells define the opposing problem. Loss of tolerance is not explained only by excessive effector-cell activation; it also reflects failure of regulatory cells to adapt to inflamed environments [11]. Treg cells are often described as preferentially reliant on oxidative metabolism and fatty acid oxidation, whereas inflammatory T cells are described as glycolytic. This distinction is useful but incomplete [12]. Treg cells must maintain suppressive function under conditions of inflammatory cytokines, altered nutrient availability, mitochondrial stress and tissue damage [13]. Their fitness depends on coordinated mitochondrial, lysosomal and nutrient-sensing pathways rather than on a single preferred fuel source [13]. Treg dysfunction may therefore partly reflect an impaired capacity to maintain suppressive function within a metabolically hostile niche.

B cells add a further layer to the pathogenic set point. In systemic autoimmunity, autoreactive B cells do more than produce antibodies. They present antigen, receive T-cell help, release cytokines, differentiate into plasmablasts or plasma cells, and generate immune complexes that amplify tissue inflammation [14]. Each of these functions imposes metabolic requirements. Activation and differentiation require increased nutrient uptake, mitochondrial function, biosynthetic metabolism and endoplasmic reticulum adaptation [14]. Long-lived antibody-producing cells may also persist in supportive niches that protect them from transient inflammatory suppression [27]. B-cell metabolism may therefore contribute to disease persistence by supporting antigen presentation, lymphoid organization, autoantibody production, and tissue inflammation.

Myeloid cells are particularly important because they convert tissue stress into inflammatory output. Monocytes, macrophages and dendritic cells sense hypoxia, dying cells, immune complexes, microbial products and lipid mediators [8]. Their metabolic programs influence cytokine production, inflammasome activity, antigen presentation, phagocytosis and tissue repair [8,17]. Glycolytic activation, mitochondrial stress, altered tricarboxylic-acid-cycle activity and lipid handling can each promote inflammatory or reparative functions depending on context [8,17]. In chronic autoimmune disease, the problem may be not only inappropriate activation of myeloid cells, but persistence of myeloid states that remain poised for exaggerated responses after the original trigger has waned [21].

These cellular circuits should not be viewed in isolation. Effector T cells, Treg cells, B cells and myeloid cells compete for nutrients, exchange metabolites, respond to shared tissue-derived cues and reshape one another's function. A glycolytic macrophage can influence T-cell differentiation through cytokines and metabolite availability. Activated T cells can support B-cell differentiation and alter stromal organization. B cells and immune complexes can reinforce myeloid activation. Impaired Treg adaptation can allow these loops to continue unchecked. Within the proposed framework, these interactions are hypothesized to promote a relatively stable pathogenic state when they become mutually reinforcing and difficult to reverse.

This view has therapeutic implications. Inhibiting a metabolic pathway may reduce one pathogenic function while impairing a regulatory or reparative function elsewhere. Conversely, a therapy not designed as a metabolic intervention may still alter immunometabolic states if it disrupts cellular feedback loops that contribute to disease persistence. The challenge is to identify which metabolic circuits dominate in each patient, tissue and disease stage, and which interventions can shift the system toward tolerance without broadly compromising host defense.

## 5 Tissue metabolic niches as potential regulators of autoimmune states

Autoimmune inflammation is experienced by immune cells within tissues, not in isolation. This distinction matters because tissues are metabolically structured environments [11,15]. Oxygen tension, nutrient availability, extracellular pH, lipid composition, stromal-cell activity, microbial products, mitochondrial stress and tissue damage vary across organs and change further during inflammation. These conditions can influence which immune-cell metabolic programs are sustainable, which functions are favored and whether inflammatory responses resolve or persist [11,15]. Tissue metabolic niches may therefore actively influence autoimmune inflammation rather than merely serving as passive sites of immune-cell infiltration.

Rheumatoid arthritis provides a useful example [28,29]. The inflamed synovium is characterized by cellular expansion, vascular remodeling, relative hypoxia, altered glucose handling and accumulation of metabolites such as lactate. These features may not merely accompany inflammation. They can influence fibroblast-like synoviocytes, macrophages, endothelial cells and lymphocytes, thereby reinforcing the local inflammatory circuit. In this setting, the joint may function as a metabolically altered niche that favors the persistence or reactivation of local inflammation even when systemic inflammatory markers fluctuate.

Similar principles apply to barrier tissues [30,31]. In inflammatory bowel disease, intestinal inflammation occurs within a compartment shaped by oxygen gradients, epithelial metabolism, microbial communities and microbial metabolites. Loss of barrier integrity changes the exchange of nutrients, antigens and microbial products between the lumen and the mucosal immune system. Short-chain fatty acids, bile-acid derivatives, tryptophan metabolites and other microbial products can influence Treg and Th17 differentiation, epithelial repair and myeloid-cell activation. Within the proposed framework, persistent intestinal inflammation may reflect not only immune activation, but also reciprocal disruption of epithelial, microbial, and immune metabolic homeostasis.

Systemic lupus erythematosus illustrates how systemic immune dysregulation can become organ-specific metabolic injury. Mitochondrial dysfunction, oxidative stress, type I interferon signaling, immune-complex deposition and complement activation may converge differently in blood, kidney, skin and other target tissues [[32], [33], [34]]. In lupus nephritis, local tissue injury and inflammatory infiltration may create a renal environment in which immune activation, cellular stress and impaired repair reinforce one another [32,34]. Because much of the current immunometabolic evidence in SLE comes from circulating immune cells, direct tissue-level measurements will be important for understanding organ damage and relapse.

In the central nervous system, metabolic niche effects are difficult to separate from tissue function. Multiple sclerosis and related neuroinflammatory disorders involve immune infiltration, myelin injury, glial activation and altered lipid handling [35,36]. Because myelin is lipid rich, and remyelination depends on coordinated lipid metabolism, mitochondrial function and glial support, neuroinflammation cannot be understood only as lymphocyte entry into the CNS [[35], [36], [37]]. Disturbed lipid recycling, cholesterol handling, oxidative stress and microglial or macrophage metabolism may help determine whether inflammatory lesions resolve, remain chronically active or fail to repair.

The same logic can be extended to skin, muscle, salivary glands and other autoimmune target tissues. Local stromal cells, epithelial cells, endothelial cells, fibroblasts and resident macrophages shape immune-cell access to nutrients and survival signals [11,15]. They also respond to immune-derived cytokines by changing their own metabolic state. A pathogenic tissue niche may therefore represent more than an abnormal environment imposed on immune cells; it is continuously remodeled by the immune response itself. Once this feedback becomes established, suppressing one inflammatory mediator may not be sufficient to restore the tissue to its pre-inflammatory state [23].

These observations help explain why systemic biomarkers may incompletely capture autoimmune disease biology. Circulating cytokines, metabolites or immune-cell phenotypes can be informative, but they may miss compartmentalized abnormalities in synovium, gut mucosa, kidney, skin or CNS. A patient may appear clinically improved while residual tissue hypoxia, stromal activation, mitochondrial stress or metabolite gradients remain. Such residual abnormalities could contribute to a relapse-prone state by preserving local conditions that favor rapid reactivation of pathogenic immune cells.

Tissue metabolic niches may therefore be important when distinguishing inflammatory suppression from immunometabolic reset. A therapy may reduce circulating inflammation without normalizing the tissue environment that originally sustained disease. Conversely, interventions that restore barrier metabolism, resolve stromal activation, improve mitochondrial quality control, alter microbial metabolite production or remodel lipid handling may contribute to reset even if they are not classically defined as immunometabolic drugs. The clinically relevant question is whether treatment modifies tissue conditions that may support the persistence or reactivation of pathogenic immunity in patients, rather than merely altering a metabolic pathway in vitro. Table 1 summarizes how tissue metabolic niches may differ across autoimmune disease contexts.

**Table 1:** Illustrative tissue immunometabolic niches, affected immune compartments, relapse-related implications, and candidate reset-oriented strategies across autoimmune diseases.

Disease context	Dominant tissue niche	Extrinsic metabolic features	Immune compartments affected	Relapse-relevant implication	Candidate reset-oriented strategy
Rheumatoid arthritis	Inflamed synovium	Hypoxia, lactate accumulation, altered glucose handling, stromal metabolic activation, acidic microenvironment	Fibroblast-like synoviocytes, macrophages, endothelial cells, T cells, B cells	Persistent synovial niche may sustain local inflammation despite fluctuating systemic inflammatory markers	Resolve stromal activation, normalize hypoxia and lactate-rich niches, disrupt fibroblast–myeloid–lymphocyte feedback
Inflammatory bowel disease	Intestinal mucosa and epithelial barrier	Oxygen gradients, epithelial metabolic stress, microbial dysbiosis, altered short-chain fatty acids, bile-acid derivatives and tryptophan metabolites	Epithelial cells, Treg cells, Th17 cells, myeloid cells, innate lymphoid cells	Barrier dysfunction and altered microbial metabolism may preserve relapse-prone mucosal immune activation	Restore barrier metabolism, reshape microbial metabolite production, reinforce epithelial repair and mucosal tolerance
Systemic lupus erythematosus and lupus nephritis	Blood, kidney, skin and other target tissues	Mitochondrial dysfunction, oxidative stress, type I interferon signaling, immune-complex deposition, complement activation	Plasmablasts, autoreactive B cells, T cells, monocytes, tissue-infiltrating immune cells	Systemic immune dysregulation may translate into organ-specific tissue injury and incomplete repair	Reduce autoreactive memory, improve mitochondrial and redox balance, assess tissue-level immune and metabolic injury
Multiple sclerosis and neuroinflammation	Central nervous system lesions	Altered lipid handling, cholesterol recycling, oxidative stress, mitochondrial dysfunction, glial metabolic activation	T cells, B cells, microglia, macrophages, oligodendrocyte-lineage cells	Failure of lipid recycling and repair programs may favor chronic active lesions or impaired remyelination	Support lipid handling, remyelination, mitochondrial quality control and resolution of glial inflammation
Psoriasis and inflammatory skin disease	Epidermis and dermal immune niche	Keratinocyte metabolic activation, lipid remodeling, hypoxia, cytokine-driven stromal and epithelial changes	Keratinocytes, dendritic cells, macrophages, Th17 cells, tissue-resident memory T cells	Residual tissue-resident immune and epithelial programs may enable rapid lesion recurrence	Restore epithelial–immune metabolic balance, target tissue-resident memory and normalize inflammatory skin niches
Sjögren disease and glandular autoimmunity	Salivary and lacrimal glands	Epithelial stress, mitochondrial dysfunction, stromal remodeling, local nutrient and survival niches	Epithelial cells, B cells, plasma cells, T cells, resident myeloid cells	Persistent glandular niches may maintain autoreactive lymphocytes and local tissue dysfunction	Combine immune-memory targeting with tissue repair and restoration of epithelial metabolic function

Table note. Examples are illustrative rather than exhaustive. Candidate reset-oriented strategies indicate biological mechanisms that may need to be modified for durable remission; they are not intended as disease-specific treatment recommendations. CNS, central nervous system; Treg, regulatory T cell; Th17, T helper 17 cell.

## 6 Metabolites as messengers of tissue stress and immune memory

Metabolites are often treated as downstream readouts of immune activation. In autoimmune disease, this view is too limited. Many metabolites are positioned at the interface between cellular metabolism, tissue stress and immune signaling [38]. They can reflect the metabolic state of an inflamed tissue, but they may also influence immune-cell differentiation, cytokine production, chromatin structure, post-translational modification and antigenicity [16,38]. Metabolites are therefore not only biochemical intermediates; they may also act as mediators through which tissue environments influence immune-cell function and, in some settings, persistent cellular responses [21].

Lactate illustrates this shift in thinking. Traditionally regarded as the product of glycolysis under conditions of high metabolic demand or limited oxygen availability, lactate is now recognized as an immunologically active metabolite [39]. In inflamed tissues, increased glycolytic activity in immune and stromal cells can lead to local lactate accumulation [28]. This may alter extracellular pH, influence macrophage and T-cell behavior, and affect stromal-cell function. The meaning of lactate is context dependent. It may support inflammatory circuits in one tissue and contribute to regulatory or reparative functions in another [39]. Its importance lies not in being uniformly pro-inflammatory or anti-inflammatory, but in carrying information about tissue metabolic stress into cellular responses.

The emerging biology of lactylation extends this idea further. Lactate-derived lysine lactylation provides a potential route by which metabolic states can be written onto proteins, including histones. This is relevant to autoimmunity because epigenetic changes may help stabilize inflammatory phenotypes after the initiating stimulus has waned [16,21]. Recent evidence in rheumatoid arthritis has linked increased lactylation in fibroblast-like synoviocytes with histone modification and autoantibody responses against lactylated histones [16]. These observations do not prove that lactylation is a universal driver of autoimmune disease. They do, however, show how a metabolite-rich tissue niche could influence chromatin state and possibly antigenic targets, thereby connecting metabolism, stromal activation and adaptive immunity in a clinically relevant setting.

Succinate represents another form of metabolite signaling. As an intermediate of the tricarboxylic acid cycle, succinate can accumulate when mitochondrial metabolism is rewired during inflammatory activation [17]. In macrophages, succinate has been linked to stabilization of HIF-1α and increased IL-1β production, providing a mechanistic bridge between mitochondrial state and inflammatory output. This pathway is often discussed in innate immune activation, but it also has broader implications for autoimmune inflammation. If tissue stress, hypoxia or mitochondrial dysfunction promote succinate accumulation, then a metabolic intermediate could contribute to inflammatory feedback that, within the proposed framework, may reinforce a pathogenic state [17,38].

Itaconate provides an important counterpoint. Produced through the IRG1-encoded pathway in activated myeloid cells, itaconate and its derivatives have been associated with antioxidant and anti-inflammatory programs, including modulation of NRF2-dependent responses [18]. This does not mean that itaconate is simply protective in all settings. Its effects depend on dose, timing, cell type, chemical form and inflammatory context [40]. Nevertheless, itaconate is conceptually useful because it shows that inflammatory metabolism can also generate endogenous braking mechanisms. A pathogenic set point may therefore reflect not only excessive inflammatory metabolites, but also failure, insufficiency or mistiming of counter-regulatory metabolic signals.

Other metabolites link nutrient state to immune fate in less linear ways. Acetyl-CoA links carbon metabolism to histone acetylation, whereas one-carbon metabolism provides one-carbon units required for nucleotide biosynthesis and methylation reactions [41,42]. Tryptophan catabolism through kynurenine pathways can influence immune tolerance, barrier tissues and regulatory circuits [43]. Lipid mediators can shape leukocyte recruitment, resolution, macrophage polarization and tissue repair [44]. Together, these pathways form a metabolite signaling network that changes as inflammation evolves from initiation to chronicity, remission or relapse.

For the set point model, the central lesson is that metabolites are neither passive by-products nor standalone therapeutic targets. Within the proposed framework, metabolites are considered potential components of feedback networks linking immune activation with tissue stress, chromatin regulation, and memory. Testing whether a therapy produces an immunometabolic reset will require assessing not only whether inflammatory cytokines fall, but whether metabolite signaling networks and their downstream cellular effects return toward a tolerogenic state.

## 7 Metabolic memory and relapse-prone remission

Relapse after treatment reduction or withdrawal provides a clinical rationale for distinguishing inflammatory suppression from a more durable biological reset. In many autoimmune diseases, clinical remission can be achieved while therapy is maintained, yet disease activity may return after dose reduction, treatment withdrawal or a new environmental challenge [23,45]. This pattern does not necessarily mean that therapy has failed. It may instead indicate that inflammatory output was suppressed more effectively than the biological state that made inflammation likely in the first place [23].

Metabolic memory offers a framework for understanding this residual susceptibility. Previous inflammatory exposure can leave durable changes in immune cells, stromal cells or tissue-resident compartments [21,46]. These changes may involve altered mitochondrial function, redox balance, nutrient use, metabolite availability, chromatin accessibility or transcriptional responsiveness [21,46]. Such memory does not need to produce overt inflammation at rest. Such changes may lower the threshold for renewed activation when the system encounters antigen, infection, tissue injury, hormonal changes, microbial perturbation or treatment withdrawal.

Trained immunity provides one mechanism through which innate immune cells may retain inflammatory memory [21]. Monocytes, macrophages and their progenitors can undergo metabolic and epigenetic reprogramming after exposure to inflammatory stimuli. In some contexts, this enhances host defense. In others, it may promote exaggerated or inappropriate inflammatory responses. In autoimmune disease, trained myeloid states could contribute to relapse-prone remission by preserving cells that respond too strongly to secondary challenges [21]. This possibility is relevant because innate immune activation often precedes, amplifies or shapes adaptive autoimmune responses.

The bone marrow may also contribute to relapse biology. If hematopoietic stem and progenitor cells acquire inflammatory or metabolic bias, they can generate downstream myeloid cells that are already primed toward heightened responsiveness [46,47]. This idea expands autoimmune memory beyond antigen-specific lymphocyte clones. It suggests that chronic inflammation may be partly stored in the hematopoietic system as a bias in cellular output and responsiveness. Its relevance will probably vary across diseases and treatment contexts, but it offers a plausible mechanism through which peripheral inflammation can re-emerge after apparent clinical control [46,47].

Adaptive immune memory remains equally important. Autoreactive T cells, T follicular helper cells, memory B cells and long-lived plasma cells can persist after clinical improvement [26,27]. These populations may occupy survival niches and retain metabolic programs that support persistence, rapid recall responses or autoantibody production. Relapse may involve more than innate immune memory alone and could reflect interactions among persistent autoreactive lymphocytes, primed myeloid cells, and tissue environments that remain permissive for inflammation.

Tissue memory may represent another component of relapse-prone remission [11]. Inflamed tissues may not return completely to their pre-disease state after symptoms improve. Fibroblasts, endothelial cells, epithelial cells, glial cells or tissue-resident macrophages may retain altered transcriptional and metabolic programs [11,15]. Residual hypoxia, stromal activation, mitochondrial stress, lipid remodeling or barrier dysfunction can create a niche in which pathogenic immune cells are more easily reactivated.

This view reframes remission. Clinical remission is usually defined by symptoms, laboratory markers, imaging, organ function or disease activity scores. These measures are essential, but they may not reveal whether pathogenic immunometabolic memory has been erased. A patient may therefore be clinically quiescent while retaining biological features associated with relapse risk. Conversely, durable remission may require coordinated normalization across autoreactive lymphocyte pools, myeloid responsiveness, regulatory-cell fitness, tissue metabolic niches and metabolite signaling.

**Fig. 2:**
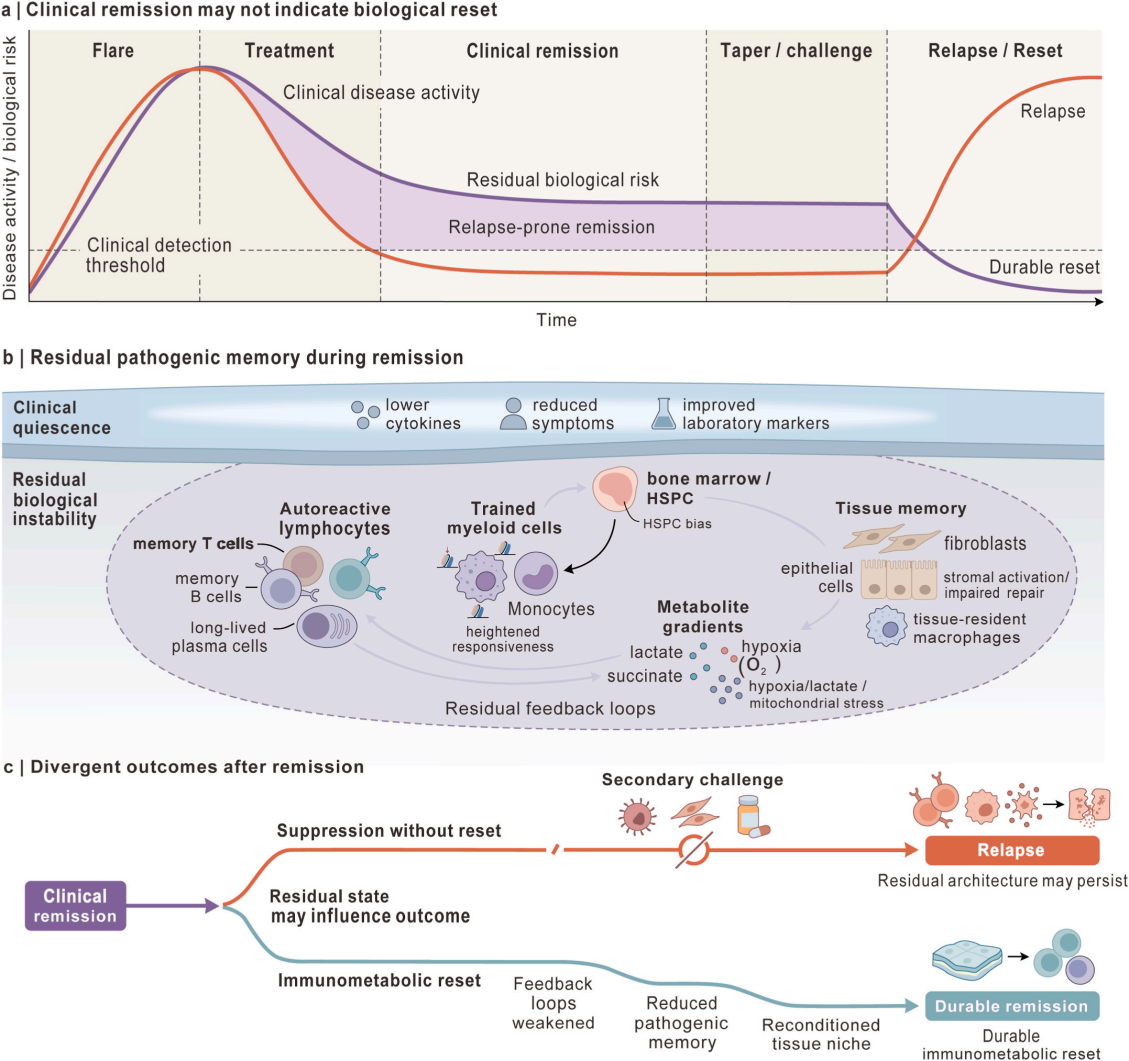
Conceptual model of relapse-prone remission arising from residual immunometabolic instability. (a) Clinical disease activity may fall below the detection threshold during treatment, while residual biological risk persists and may become apparent after treatment tapering or a secondary challenge. (b) Candidate sources of residual instability include autoreactive lymphocytes, trained myeloid responsiveness, hematopoietic stem and progenitor cell bias, tissue memory, and persistent metabolite gradients. (c) Suppression without reset may permit relapse, whereas evidence consistent with immunometabolic reset would include weakened feedback loops, reduced pathogenic memory, reconditioned tissue niches, and durable remission. The trajectories are schematic and are not intended to represent quantitative clinical thresholds. HSPC, hematopoietic stem and progenitor cell.

Relapse should therefore not be studied only as a failure of terminal treatment response. It should also be studied as a state-transition problem. Which components of the pathogenic set point persist during remission? Which ones predict flare after treatment withdrawal? Which therapies reduce inflammatory output without changing memory, and which therapies alter the memory architecture itself? Answering these questions will require longitudinal sampling before treatment, during remission and at relapse, ideally with paired blood and tissue analysis. Fig. 2 illustrates how clinical remission may coexist with residual biological instability and relapse risk.

## 8 From inflammatory suppression to immunometabolic reset

The distinction between inflammatory suppression and immunometabolic reset is therapeutically important. Many current treatments reduce disease activity by inhibiting cytokines, blocking signaling pathways, depleting immune-cell subsets or broadly suppressing immune activation [4,5]. These approaches have transformed the management of autoimmune disease and remain essential, particularly in organ-threatening disease. However, reduced inflammatory output does not necessarily mean that the underlying pathogenic set point has been reversed [23]. A patient may show improved symptoms, lower inflammatory markers or reduced autoantibody titers while retaining tissue niches, immune memory or metabolic programs that allow disease to recur [11,23].

Inflammatory suppression can itself involve metabolic effects. Glucocorticoids, antimetabolites, calcineurin inhibitors, JAK inhibitors, mTOR inhibitors, biologics and targeted synthetic drugs influence immune-cell function in ways that may intersect with metabolism [10,48]. Some reduce the energetic demands of activation by lowering cytokine signaling or proliferation. Others alter nutrient sensing, mitochondrial function, redox balance or biosynthetic pathways more directly [10,48]. The more relevant distinction is not whether a therapy is immunological or metabolic, but whether it produces transient control or durable changes in feedback loops that may contribute to disease persistence [11].

Metabolic modulators provide an instructive but complex example. Targeting mTOR, AMPK, glycolysis, glutamine metabolism, lipid metabolism or mitochondrial function can shift immune-cell behavior in experimental systems and, in selected settings, in patients [10,48,49]. These strategies are attractive because they address processes required for immune activation, differentiation and survival. Yet the same metabolic pathway may support both pathogenic and regulatory functions depending on cell type, tissue and disease stage. Metabolic targeting therefore requires precision rather than pathway-wide inhibition.

Within the proposed framework, evidence of therapeutic reset would require a higher standard than short-term reduction in disease activity. Such evidence would include not only reduced cytokine production or pathogenic immune-cell abundance, but also durable changes associated with a lower likelihood of reactivation. This might involve restoration of regulatory T-cell fitness, reduction of autoreactive lymphocyte persistence, reprogramming of trained myeloid states, resolution of stromal activation, normalization of tissue oxygenation or nutrient gradients, and correction of metabolite signaling networks. No single therapy is expected to accomplish all these effects in every disease. The set point framework instead asks which components of the pathogenic state are dominant in each patient and whether they have been durably altered.

Cellular therapies and profound immune-depleting strategies have brought this question into sharper focus. Hematopoietic stem cell transplantation, deep B-cell depletion and CD19-targeted CAR-T-cell therapy can induce marked clinical responses in selected severe autoimmune diseases [24,50]. Their appeal lies not only in stronger immunosuppression, but in the possibility that they may disrupt self-sustaining immune networks more fundamentally than conventional maintenance therapy [24,50]. However, whether such interventions reset immunometabolism remains incompletely defined. Durable treatment-free remission would be consistent with reset, but it is not sufficient proof. Paired analyses of lymphocyte reconstitution, myeloid responsiveness, tissue metabolism, metabolite signaling and regulatory-cell function are needed to determine what has changed.

B-cell-directed therapies illustrate the difference between depletion and reset. Removing circulating B cells may reduce antigen presentation, cytokine production and new plasmablast generation, but long-lived plasma cells, tissue-resident B cells or autoreactive memory compartments may persist [27,51]. More intensive approaches may achieve deeper depletion and immune reconstitution, yet their long-term effects on tissue niches and metabolic memory remain uncertain [51]. From an immunometabolic perspective, the key question is not only whether autoreactive B cells disappear, but whether the conditions that allowed their activation, survival and collaboration with T cells and myeloid cells have been altered.

Regulatory-cell-based therapies may represent another potential route toward reset. Low-dose IL-2, adoptive Treg therapy and engineered regulatory cells aim to restore tolerance rather than simply block inflammation [52,53]. Their success may depend on whether transferred or expanded regulatory cells can maintain metabolic fitness within inflamed tissues [13,53]. A Treg program that appears functional in blood may fail in a hypoxic, nutrient-depleted or cytokine-rich niche. Regulatory restoration is therefore inseparable from tissue metabolic context.

Tissue-directed interventions may also contribute to strategies intended to achieve durable biological reconfiguration. Therapies that improve barrier integrity, alter microbial metabolite production, reduce stromal activation, resolve fibrosis, improve mitochondrial quality control or normalize lipid handling may indirectly change immune-cell behavior [30,31,35,36]. These approaches are sometimes viewed as secondary to immune suppression, but the set point framework highlights their potential relevance to disease persistence. If tissue niches contribute to the persistence of pathogenic immunity, tissue repair and metabolic reconditioning may form part of strategies aimed at achieving durable remission.

The proposed framework does not support universal metabolic inhibition as a therapeutic objective. It should be framed as selective destabilization of pathogenic feedback loops and restoration of tolerogenic ones. Some patients may require suppression of effector-cell metabolism; others may require repair of tissue niches, removal of autoreactive memory, enhancement of regulatory metabolism or combinations of these approaches. The set point model provides a way to ask whether treatment has merely lowered inflammation or has changed the state from which inflammation emerges. Key distinctions between inflammatory suppression and immunometabolic reset are summarized in Table 2.

**Table 2:** Proposed criteria and evidence requirements for distinguishing inflammatory suppression from immunometabolic reset.

Dimension	Inflammatory suppression	Immunometabolic reset	Evidence required
Clinical state	Reduced symptoms, disease activity scores or organ inflammation during treatment	Durable remission with reduced relapse propensity, ideally after dose reduction or treatment withdrawal	Longitudinal clinical follow-up, flare assessment and treatment-tapering data where appropriate
Inflammatory mediators	Lower cytokines, acute-phase reactants or transcriptional inflammatory scores	Sustained normalization of inflammatory output together with reduced reactivation potential	Serial cytokine, transcriptomic and functional immune assays
Effector immune cells	Reduced activation, proliferation or abundance of pathogenic immune cells	Loss, reprogramming or functional restraint of pathogenic effector-cell states	Single-cell phenotyping, antigen-specific assays and functional stimulation studies
Regulatory compartment	Partial recovery of regulatory signals or cell frequencies	Restoration of regulatory-cell fitness and suppressive function within inflamed or previously inflamed tissues	Treg and tolerogenic myeloid functional assays, preferably paired with tissue context
Tissue niche	Persistent hypoxia, stromal activation, barrier dysfunction or metabolic stress may remain	Reconditioning of tissue oxygenation, nutrient gradients, stromal state, barrier function and repair programs	Spatial profiling, imaging, tissue metabolomics and histological assessment
Metabolite signaling	Disease-associated metabolites may decrease without clear restoration of network balance	Metabolite signaling shifts toward tolerogenic, reparative or homeostatic states	Targeted and untargeted metabolomics, spatial metabolomics and pathway-level interpretation
Immune memory	Autoreactive lymphocytes, long-lived plasma cells or trained myeloid states may persist	Reduced pathogenic memory or altered memory architecture that raises the threshold for relapse	Memory B-cell, plasma-cell, T-cell, myeloid and hematopoietic profiling
Metabolic function	Inferred from changes in pathway-associated genes or circulating metabolites	Demonstrated change in pathway activity and cellular metabolic fitness	Flux assays, mitochondrial measurements, isotope tracing or ex vivo functional testing
Interpretation	Disease output is controlled	Integrated evidence is consistent with durable alteration of biological processes associated with disease persistence	Integrated clinical, cellular, tissue and metabolic evidence
Therapeutic implication	Continue, taper or switch anti-inflammatory therapy based on disease activity	Match therapy to dominant set point drivers, including effector-cell, regulatory-cell, tissue-niche or memory-dominant states	Prospective validation in stratified cohorts and clinical trials

Table note. Inflammatory suppression and immunometabolic reset are not mutually exclusive. Effective therapy may begin with suppression, but evidence of reset requires durable changes across immune, tissue, metabolic and memory compartments. The proposed criteria are conceptual and should be tested prospectively in disease-specific cohorts.

## 9 Measuring and targeting immunometabolic set points

For the immunometabolic set point to function as a testable framework, it must be linked to measurable biological features. This does not mean that one assay will define a patient's set point. A stable autoimmune state is unlikely to be captured by a single circulating metabolite, cytokine or transcriptomic score. Rather, it should be inferred from coordinated patterns across immune-cell states, tissue metabolic niches, metabolite signaling, cellular memory and clinical behavior over time. The methodological challenge is to move from static and compartment-limited measurements toward longitudinal and spatially resolved profiling of autoimmune metabolism.

Peripheral blood remains the most accessible starting point. Flow cytometry, mass cytometry, single-cell RNA sequencing, CITE-seq, epigenomic profiling and serum or plasma metabolomics can identify immune-cell phenotypes, activation states and systemic metabolic signatures [54]. These approaches are useful for patient stratification and treatment monitoring, especially when repeated sampling is required. However, blood is an imperfect proxy for tissue disease. Circulating cells may not reflect the metabolic state of synovium, kidney, gut mucosa, skin or central nervous system lesions [11,15]. A patient can show normalized systemic markers while tissue-resident immune cells, stromal cells or local metabolite gradients remain abnormal.

Single-cell technologies have substantially advanced the characterization of cellular heterogeneity in autoimmune disease, including the identification of disease-associated immune and stromal states within affected tissues [55,56]. However, transcriptomic profiles provide only indirect evidence of metabolic activity. Expression of metabolic enzymes, transporters, and pathway-associated genes may suggest altered pathway use, but transcript abundance is not equivalent to metabolic flux, which also depends on substrate availability, enzyme activity, post-translational regulation, mitochondrial function, and cellular context [57]. Transcriptomic metabolic inference should therefore be regarded as a hypothesis-generating approach rather than a direct measurement of immunometabolic function.

Metabolomics provides a more direct view of biochemical state, but conventional bulk metabolomics also has limitations. Serum, plasma or homogenized tissue measurements average signals across multiple cell types and compartments [15,25]. They may identify disease-associated metabolites, but cannot determine which cells produce them, which cells sense them, or whether they are causal, compensatory or downstream consequences of inflammation. This distinction is especially important for metabolites whose effects depend strongly on cellular and spatial context [58].

Spatial technologies may be particularly informative for testing the set point model. Mass spectrometry imaging, spatial metabolomics, imaging mass cytometry, multiplex immunofluorescence, spatial transcriptomics and spatial proteomics can map immune cells, stromal cells and metabolites within intact tissue architecture. These approaches can show whether pathogenic immune states are localized near hypoxic zones, activated fibroblasts, damaged epithelium, lipid-rich lesions or metabolite-enriched niches. They can also reveal whether therapy normalizes tissue organization or merely reduces immune-cell abundance.

Metabolic flux measurements add another layer of information. Stable isotope tracing can distinguish metabolite abundance from pathway activity by following labeled nutrients through metabolic networks. In autoimmune disease, flux-based approaches could help determine whether effector cells, regulatory cells, myeloid cells or stromal cells use glucose, glutamine, fatty acids or other substrates differently during flare, remission and relapse. Although routine isotope tracing in patients remains technically difficult, ex vivo tissue culture, organoids, explants and carefully designed clinical sampling may make selected applications feasible.

The most informative studies will probably be longitudinal. A single baseline sample cannot define whether a patient is moving toward suppression, relapse or reset [23]. Samples should be collected before treatment, during early response, at clinical remission, after dose reduction where appropriate, and at relapse if it occurs. This design would allow investigators to ask whether residual immunometabolic abnormalities during remission predict future flare [23]. It would also help distinguish biomarkers of current inflammation from markers of relapse-prone biological memory [8,23].

A practical biomarker framework should separate three questions. First, what reflects current inflammatory burden? Second, what predicts relapse after apparent remission? Third, what indicates durable reset? These questions are related but not identical. A marker that tracks disease activity may fail to predict relapse. A relapse predictor may not reveal the causal mechanism. A reset marker should ideally show persistent normalization after treatment withdrawal or reduced treatment intensity. Without this distinction, studies risk confusing short-term suppression with long-term state change.

For clinical translation, the measurement strategy must remain realistic. Deep spatial and flux-based profiling will not be applied to every patient in routine care. A more plausible route is staged discovery. High-dimensional tissue and metabolic studies can define candidate set point signatures in well-characterized cohorts. These signatures can then be reduced to practical blood-based, imaging-based or biopsy-based assays for validation. If prospectively validated, immunometabolic profiling could ultimately inform therapeutic decisions: whether to intensify inflammatory suppression, restore regulatory function, target autoreactive memory, repair tissue niches or combine these approaches.

**Fig. 3:**
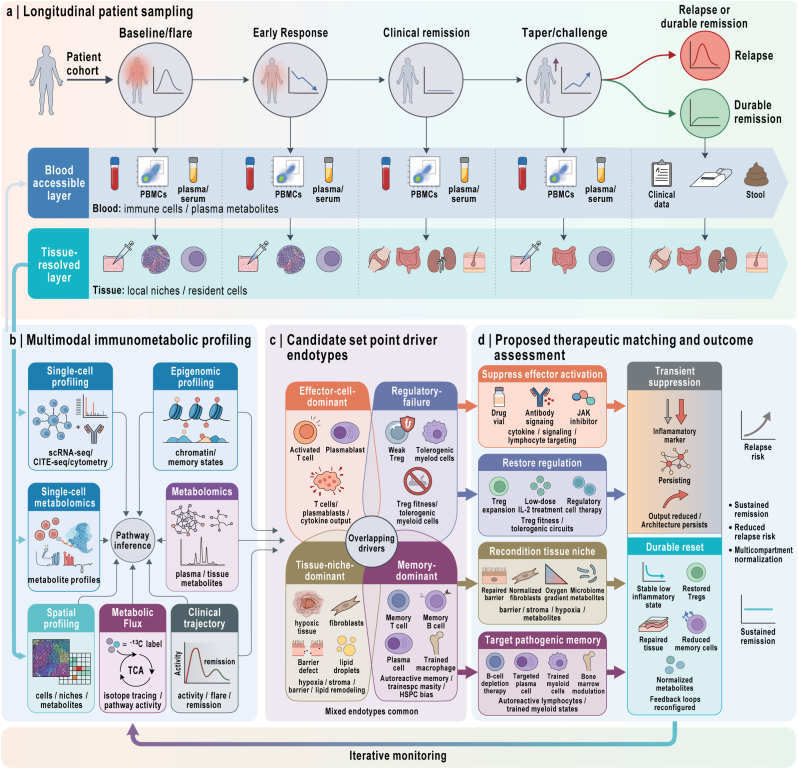
Proposed longitudinal precision-immunometabolism workflow for measuring and targeting autoimmune set points. (a) Longitudinal sampling across baseline or flare, early response, clinical remission, treatment tapering or challenge, and subsequent relapse or durable remission, with paired blood and disease-relevant tissue sampling where feasible. (b) Integration of single-cell, epigenomic, metabolomic, spatial, and metabolic-flux measurements with clinical trajectories to infer candidate biological pathways. (c) Candidate set point driver endotypes include effector-cell-dominant, regulatory-failure, tissue-niche-dominant, and memory-dominant states, which may overlap within individual patients. (d) These candidate endotypes could inform hypothesis-driven therapeutic matching and longitudinal outcome assessment to distinguish transient inflammatory suppression from evidence consistent with durable immunometabolic reset. The workflow is conceptual and requires prospective validation. CITE-seq, cellular indexing of transcriptomes and epitopes by sequencing; HSPC, hematopoietic stem and progenitor cell; IL-2, interleukin-2; JAK, Janus kinase; PBMC, peripheral blood mononuclear cell; scRNA-seq, single-cell RNA sequencing; TCA, tricarboxylic acid cycle; Treg, regulatory T cell.

The set point framework suggests that a one-size-fits-all approach to immunometabolic therapy may be inadequate. Patients with the same diagnosis may differ in the candidate mechanisms contributing to disease persistence. Some may have an effector-cell-dominant state, characterized by persistent inflammatory T-cell or plasmablast activity. Others may have a regulatory-failure state, in which Treg cells or tolerogenic myeloid populations cannot maintain function in inflamed tissue [13]. A tissue-niche-dominant state may be marked by hypoxia, stromal activation, lipid remodeling, fibrosis, epithelial barrier dysfunction or altered microbial metabolism. A memory-dominant state may involve autoreactive lymphocyte persistence, trained myeloid responsiveness or hematopoietic bias [21]. These provisional categories are not mutually exclusive but may provide a basis for moving beyond disease labels alone. Fig. 3 summarizes a proposed clinical translation pathway in which longitudinal blood and tissue sampling, multimodal immunometabolic profiling and dominant set point driver endotypes are linked to therapeutic matching and outcome assessment.

## 10 Cautions and future direction

Several cautions are necessary. First, immunometabolic reset should not be equated with permanent cure. Autoimmune susceptibility may persist through genetics, environmental exposures, antigenic triggers and tissue vulnerabilities. Second, metabolic pathways are shared across host defense, tissue repair and immune regulation, so broad metabolic inhibition may cause toxicity or increase infection risk. Third, evidence from animal models and ex vivo systems may not translate directly to patients, especially when tissue architecture and chronic disease history are absent. Finally, durable treatment-free remission in selected patients is compatible with reset, but mechanism-specific evidence is still required.

Several methodological challenges further complicate validation of the proposed framework. First, no standardized operational definition or quantitative threshold currently exists for assigning an immunometabolic set point. Second, associations among immune-cell states, tissue conditions, metabolites, and clinical relapse do not by themselves establish causal or self-reinforcing relationships. Third, repeated sampling of disease-relevant tissues is often impractical, and peripheral blood measurements may not capture compartmentalized tissue biology. Fourth, heterogeneity across autoimmune diseases, target organs, treatment histories, and disease stages may limit the generalizability of a single framework. In addition, findings from animal models and ex vivo systems may not reproduce the chronic, multicompartment biology of human autoimmune disease. Prospective studies are therefore required to determine whether coordinated immunometabolic changes predict relapse or durable treatment-free remission beyond conventional clinical and inflammatory measures.

The next phase of the field should focus on testable definitions. What minimum biological criteria distinguish suppression from reset? Which biomarkers predict relapse after apparent remission? Which tissue or blood signatures show that regulatory metabolism has recovered? Which interventions alter trained immunity, autoreactive memory or tissue metabolic niches in humans? These questions should be embedded into clinical trials rather than addressed retrospectively. Ultimately, precision immunometabolic reset is not a proposal to replace existing autoimmune therapies. It is a proposal to understand why some treatments produce transient control whereas others may permit durable remission, and why patients with the same diagnosis may require different routes back to tolerance. Its value will depend on whether it helps identify measurable relapse-prone states and guides interventions that shift patients from inflammatory control toward durable immune tolerance.

## Funding

This study was funded by Scientific Research Project of 10.13039/100017962Jiangsu Provincial Health Commission (K2024084, JSZJ20241204). The funder had no role in the conception of the Review, literature evaluation, manuscript preparation, or the decision to submit the article.

## CRediT authorship contribution statement

**Xiao Yu:** Visualization, Writing – original draft, Writing – review & editing. **Yidan Zhang:** Supervision, Validation, Writing – review & editing. **Anquan Shang:** Investigation, Supervision, Writing – review & editing.

## Declaration of competing interest

The authors declare that they have no known competing financial interests or personal relationships that could have appeared to influence the work reported in this paper.

## Data Availability

No data was used for the research described in the article.

## Abbreviations

Acetyl-CoA: acetyl coenzyme A
AMPK: AMP-activated protein kinase
CAR-T: chimeric antigen receptor T cell
CD19: cluster of differentiation 19
CITE-seq: cellular indexing of transcriptomes and epitopes by sequencing
CNS: central nervous system
FA: fatty acid
HIF-1α: hypoxia-inducible factor 1 alpha
HSPC: hematopoietic stem and progenitor cell
IL: interleukin
IRG1: immune-responsive gene 1
JAK: Janus kinase
mTOR: mechanistic target of rapamycin
NRF2: nuclear factor erythroid 2-related factor 2
OXPHOS: oxidative phosphorylation
PBMC: peripheral blood mononuclear cell
RNA: ribonucleic acid
scRNA-seq: single-cell RNA sequencing
SLE: systemic lupus erythematosus
TCA: tricarboxylic acid
Th17: T helper 17 cell
Treg: regulatory T cell
